# The Preoperative Neutrophil-To-Lymphocyte Ratio Is a Novel Immune Parameter for the Prognosis of Esophageal Basaloid Squamous Cell Carcinoma

**DOI:** 10.1371/journal.pone.0168299

**Published:** 2016-12-13

**Authors:** Qin Xiao, Baihua Zhang, Xiang Deng, Jie Wu, Hui Wang, Yonggang Wang, Wenxiang Wang

**Affiliations:** 1 Key Laboratory of Translational Radiation Oncology, Hunan Province, Department of Radiation Oncology, Hunan Cancer Hospital, The Affiliated Cancer Hospital of Xiangya School of Medicine, Central South University, Changsha, Hunan Province, China; 2 The 2nd Department of Thoracic Surgery, Hunan Cancer Hospital, The Affiliated Cancer Hospital of Xiangya School of Medicine, Central South University, Changsha, Hunan Province, China; 3 Department of Thoracic Surgery, Cancer Hospital and Institute, Chinese Academy of Medical Sciences and Peking Union Medical College, Beijing, China; University of Michigan, UNITED STATES

## Abstract

**Background:**

The pretreatment neutrophil-to-lymphocyte ratio (NLR) is an independent predictor of prognosis in various malignancies, but its predictive capacity in basaloid squamous cell carcinoma of the esophagus (BSCCE) remains unclear. We aim to determine the value of the inflammation-related factors, including the NLR, neutrophil-to-monocyte ratio (NMR), and albumin levels, in predicting BSCCE prognosis.

**Methods:**

We retrospectively analyzed the records of 121 patients with pathologically diagnosed BSCCE that underwent a curative esophagectomy from January 2007 to December 2014. Univariate and multivariate analyses were used to identify prognostic factors for overall survival (OS) and recurrence-free survival (RFS).

**Results:**

The preoperative NLR was correlated with the tumor length and NMR. In OS univariate analyses, a high NLR (>1.77), high NMR (>12.31), and low albumin (≤40.0 g/L) level were significantly associated with a poorer survival in BSCCE. The median OS was significantly greater in low NLR (≤1.77) than in the high NLR (>1.77) patients (51.0 vs. 31.0 months; P = 0.008). In multivariate analyses, only the NLR was an independent prognostic factor for OS (hazard ratio (HR), 2.030; 95% confidence interval (CI), 1.262–3.264; P = 0.003). A high NLR was also an independent predictor of a poorer RFS in BSCCE (HR, 2.222; 95% CI, 1.407–3.508; P = 0.001); the median RFS for low (≤1.77) and high (> 1.77) NLR patients was 44.0 months and 14.0 months, respectively. NLR remained a strong prognostic indicator for OS in stage I/II patients and a preoperative NLR>1.77 was predictive of a poor RFS in both stage I/II and stage III patients.

**Conclusions:**

We show that the preoperative NLR, a convenient and cost-effective biomarker, may serve as a prognostic indicator for BSCCE patients following curative surgery.

## Introduction

Basaloid squamous cell carcinoma, which was first reported in 1986, is a specific subtype of squamous cell carcinoma (SCC) that principally occurs in the upper aerodigestive tract[1–3]. Basaloid squamous cell carcinoma of the esophagus (BSCCE) is a rare malignancy, and only a few studies with comparatively small samples have been reported in the literature [2–6]. According to previous reports[3, 5, 6], surgical resection is the best treatment option for BSCCE patients with localized lesions, but patient prognosis remains far from satisfactory. Clinicopathological factors, such as tumor size, location, tumor-node-metastasis(TNM) stage, and CK903 and CK14 expression, have all been reported to be associated with the long-term survival of BSCCE after curative surgery [3, 5, 6]. However, the prognostic value of these factors is limited, and only a small part of the prognostic heterogeneity is reflected for BSCCE. Therefore, it is important to search for more effective preoperative biomarkers.

Immunotherapy with anti-programmed death-1 or anti-programmed death ligand-1 antibodies has shown promising results in a number of malignant tumors[7, 8]. Thus, increasing attention is being paid to the immunity status and inflammatory microenvironment of patients; these play an important role in the development of various carcinomas, including esophageal carcinoma. A number of studies in different carcinoma types have shown that the density of tumor infiltrating leukocytes, including neutrophils, macrophages, CD8^+^ T lymphocytes, and Foxp3^+^ T lymphocytes, can be predictive of survival [9–11]. Peripheral blood leukocytes, including lymphocytes, monocytes, and the neutrophil-to-lymphocyte ratio (NLR), which reflects the tumor infiltrating leukocyte status to an extent, have also been shown to correlate with oncological outcomes[12–14].

Previous studies have demonstrated that patients’ pretreatment NLR is an independent predictor of long-term survival in patients with esophageal SCC and adenocarcinoma[13–16]. However, BSCCE is a specific subtype of esophageal SCC, and it has its own biological behavior and molecular features[2–4, 17]. To the best of our knowledge, there are few published reports regarding the prognostic value of the NLR in BSCCE; herein, we report our findings from a retrospective analysis of the value of preoperative peripheral blood leukocyte levels on BSCCE prognosis.

## Materials and Methods

### Study population

Patients with pathologically diagnosed BSCCE who underwent a curative esophagectomy at the Hunan Cancer Hospital or Chinese Academy of Medical Sciences Cancer Hospital from January 2007 to December 2014 were enrolled in this retrospective analysis. The study protocol was approved by the Ethics Committee of the Hunan Cancer Hospital and Chinese Academy of Medical Sciences Cancer Hospital. The study was conducted in accordance with the principles expressed in the Delclaration of Helsinki. Written informed consent was achieved at the beginning of treatment from all patients.

All patients included in the study had: 1) detailed medical histories collected and physical examinations performed prior to treatment; 2) complete blood cell counts, biochemical analyses, chest radiographs, a barium meal, computed tomography (CT) scans of the chest, and Doppler ultrasound or CT examinations of the upper abdomen completed as part of the clinical staging evaluation; 3) received curative resection as the initial treatment and no preoperative anticancer therapy; and 4) a pathological diagnosis of BSCCE confirmed when surgical specimens were re-checked by expert pathologists according to the World Health Organization classification criteria[18]. Immunohistochemical staining was also performed if necessary. Patients with acute inflammation or hematological disease prior to treatment were excluded. Patients who did not achieve curative resection or who died of postoperative complications in the 30 days after surgery were also excluded.

Clinical data for each patient included in the study, including their gender, age, preoperative complete blood cell count, albumin, and pathological parameters, were retrospectively reviewed. The absolute albumin and peripheral blood lymphocyte, neutrophil, and monocyte counts were taken from blood examinations in one week prior to surgery.

### Treatment and follow-up

All patients underwent a curative esophagectomy that used the stomach as an esophageal substitute. The middle and lower mediastinal, paracardial, superior gastric, and left gastric artery lymph nodes were routinely dissected. The surgical procedure used has previously been described [6]. Follow-up information was collected via regular outpatient clinics, telephone call, letters, and e-mails. Thoracic CT scans and Doppler ultrasound or CT examinations of the upper abdomen were performed every 3 months during the first 2 years after surgery and every 6 months thereafter. If recurrence was suspected, contrast-enhanced CT or magnetic resonance imaging was performed for confirmation. In cases of recurrence, patients received further treatment, including radiation therapy, chemotherapy, and secondary surgery, depending on the specific patient’s situation.

### Statistical analysis

Data were analyzed using Statistical Package for Social Sciences version 17.0 software (SPSS Inc., Chicago, IL, USA). The NLR was obtained by dividing the neutrophil count by the lymphocyte count, and the neutrophil-to-monocyte ratio (NMR) was obtained by dividing the neutrophil count by the monocyte count. The optimal cutoff values, defined as the point with maximal sensitivity and specificity, for the preoperative NLR, NMR, and albumin levels were calculated using receiver operating curve (ROC) analysis. Data are expressed as the mean and standard deviation for dichotomous variables, or median and range for continuous variables. Clinicopathological differences between groups were assessed using the chi-square (X^2^) test or *t* test. Overall survival (OS), the primary endpoint of the analysis, was estimated using the Kaplan-Meier method; the initial treatment date was the starting point and death or last follow-up was the end. Survival curves were compared using the log-rank test. The secondary endpoint was recurrence-free survival (RFS), which was calculated from the date of initial treatment to the date of recurrence or last follow-up. Variables that were statistically significant in the univariate analysis were included in a Cox proportional hazards regression model to determine independent prognostic factors. A two-sided probability value (P value) of less than 0.05 was considered statistically significant.

## Results

### Clinicopathological characteristics

**Fig 1:**
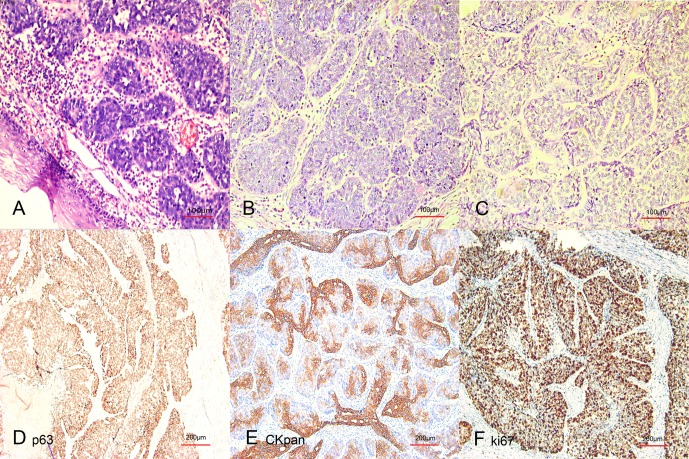
Typical microscopic features of basaloid squamous cell carcinoma of the esophagus (BSCCE). (A,B,&C) The typical features were a nesting, lobular, or trabecular arrangement of basaloid cells with comedo-type necrotic foci and small cystic spaces in the tumor center. Immunohistochemical staining using p63 (D), pan-cytokeratin(E), and Ki-67 (F) antibodies was positive.

There were 121 patients enrolled in the study (106 men and 15 women) and the median age was 62 years (range 30–76 years). The patients’ characteristics are summarized in Table 1 (data available in S1 File). Pathological examinations indicated that BSCCE was characterized by nesting, lobular, or trabecular arrays of basaloid cells with scant cytoplasm and hyperchromatic nuclei, and this was accompanied by comedo necrotic foci and small cystic spaces filled with mucinous material in the center of the tumor (Fig 1). Immunohistochemical staining was performed in 53 patients: some positive staining was observed in 26/36 patients analyzed for cytokeratin subtype expression (CK5/6, CK8, CK18, CKpan); 26/27 patients stained positive for AE1/AE3; 21/25 patients stained positive for bcl-2; 27/27 patients stained positive for CK34βE; 6/6 patients stained positive for p63; 5/8 patients stained positive for NSE; and 0/29 patients stained positive for chra.

**Table 1:** Correlation between clinicopathological characteristics and the neutrophil-to-lymphocyte ratio in basaloid squamous cell carcinoma of the esophagus

		No. of patient (n = 121)	Groups, No.		P value
			NLR≤1.77 (n = 63)	NLR>1.77 (n = 58)	
**Age (years old)**	≤60	57	32	25	0.397
	>60	64	31	33	
**Gender**	Male	106	55	51	0.916
	Female	15	8	7	
**Weight loss**	Yes	28	14	14	0.803
	No	93	49	44	
**Tumor location**	Upper	14	9	5	0.622
	Middle	75	38	37	
	Lower	32	16	16	
**Tumor length (cm)**	≤5.00	68	41	27	0.040
	>5.00	53	22	31	
**Pathological T stage**	T1/2	50	27	23	0.721
	T3/4	71	36	35	
**Pathological N stage**	N-	74	39	35	0.860
	N+	47	24	24	
**Pathological TNM**	I	25	12	13	0.841
**stage**	II	51	28	23	
	III	45	23	22	
**Lymphocyte count**	≤2.13	63	22	41	<0.001
**(×10**^**9**^**/L)**	>2.13	58	41	17	
**Neutrophil count**	≤3.74	70	51	19	<0.001
**(×10**^**9**^**/L)**	>3.74	51	12	39	
**Monocyte count**	≤0.27	52	26	26	0.693
**(×10**^**9**^**/L)**	>0.27	69	37	32	
**NMR**	≤12.31	75	49	26	<0.001
	>12.31	46	14	32	
**Albumin (g/L)**	≤40.0	46	24	22	0.985
	>40.0	75	39	36	

BSCCE: basaloid squamous cell carcinoma of the esophagus; NLR: neutrophil-to-lymphocyte ratio; NMR: neutrophil-to-monocyte ratio.

Radical resection was achieved in all patients. Postoperative complications occurred in 12 patients (9.9%), and no deaths occurred in the 30 days after surgery. By August 2016, the median duration of follow-up was 28.0 months (range 1–102 months), and 8 patients had been lost to follow-up. Cancer recurrence had occurred in 79 patients, 68 patients had died from causes secondary to BSCCE progression, and 5 patients had died from other or unknown causes.

### Determination of the best NLR and NMR cutoff values

According to ROC curve analysis, the best cutoff value for the NLR for operative prognosis was 1.77. Using this NLR cutoff value, the area under the curve (AUC) was 0.621 (95% confidence interval (CI), 0.519–0.723; P = 0.025). Patients were categorized into either low (≤ 1.77; n = 63) or high (>1.77; n = 58) NLR groups. The best cutoff value for the NMR for operative prognosis was 12.31; using this value, the AUC was 0.591 (95% CI, 0.488–0.693; P = 0.093). Patients were categorized into low (≤12.31; n = 75) and high (>12.31; n = 46) NMR groups. Similarly, the optimal cutoff value for preoperative albumin for operative prognosis was 40.0 g/L, and patients were categorized into low (≤40.0 g/L; n = 46) and high (>40.0 g/L; n = 75) albumin groups.

### Correlation between the NLR and clinicopathological features

As shown in Table 1, the relationship between the preoperative NLR and clinicopathological features was analyzed. A significant correlation between the NLR and absolute lymphocyte and neutrophil counts was observed (P<0.001). In the high NLR group, more patients had a tumor length >5.00 cm than that in the low NLR group (P = 0.040). Patients with an NLR ≤1.77 tended to have a lower NMR (P<0.001). No significant correlation between the NLR and other clinicopathological features, such as age, gender, weight loss, tumor location, pathological N, pathological T, TNM stage, and preoperative albumin, was identified in the analysis.

### OS analysis

**Fig 2:**
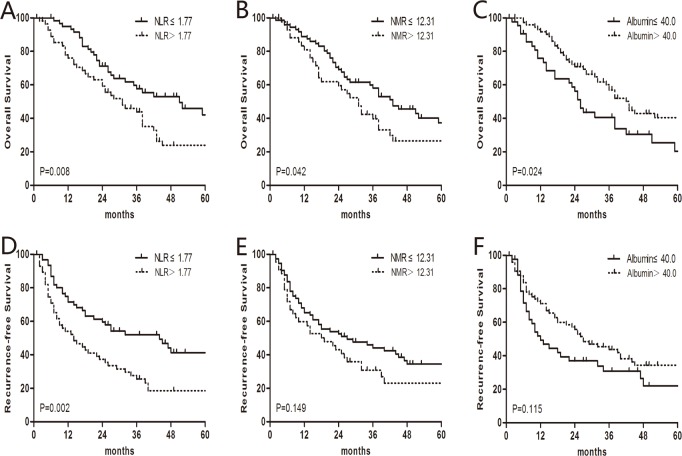
Univariate analysis of overall survival (OS) and recurrence-free survival (RFS). (A) The median OS of the low neutrophil–to–lymphocyte ratio (NLR; ≤1.77) group was 51.0 months, which was significantly longer than the 31.0 months observed in the high NLR (>1.77) group. (B) A significant difference in median OS was also observed between the low (≤12.31) neutrophil–to–monocyte ratio (NMR) group and high (>12.31) NMR group (43.0 vs. 31.0 months). (C) The median OS of the low (≤40.0 g/L) albumin groupwas 26.0 months, which was shorter than the 42.0 months observed in the high (>40.0 g/L) albumin group. (D) The median recurrence-free survival (RFS) was 44.0 months for the low (≤1.77) NLR group and 14.0 months for the high (> 1.77) NLR group. The median RFS in patients with a high NMR or albumin level was numerically, but not significantly, greater than patients with a low NMR (E) or albumin level (F).

**Fig 3:**
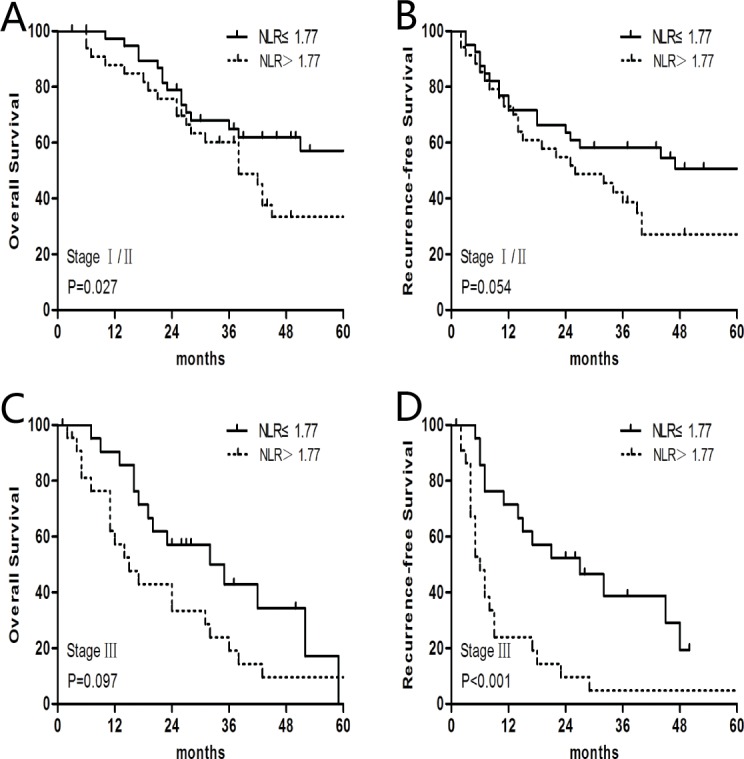
Further stratified analysis of NLR for overall survival (OS) and recurrence-free survival (RFS) in patients with different stages. (A) In stage I/II patients, the median OS in the low NLR (≤1.77; n = 40) group was 144.0 months, which was significantly longer than the 38.0 months observed in the high NLR (>1.77; n = 36) group. (B) The median RFS of the low NLR (≤1.77; n = 40) group was 105.0 months, which was longer than the 26.0 months observed in the high NLR (>1.77; n = 36) group. (C) In stage III patients, the median OS in the low NLR (≤1.77; n = 23) group was 33.5 months, which is numerically but not significantly longer than the 15.0 months observed in the high NLR (>1.77; n = 22) group.(D) The median RFS in the low NLR (≤1.77; n = 23) group was 27.0 months, which is significantly longer than the 6.0 months observed in the high NLR (>1.77; n = 22) group.

In the OS univariate analysis, the 1-, 3-, and 5-year OS rates were 84.5%, 50.1%, and 32.9%. The median OS was 38.0 months. In order to determine the optimal peripheral blood prognostic biomarkers for BSCCE, the preoperative NLR, NMR, and albumin levels were analyzed (Table 2). In the low NLR (≤1.77) group, the median OS was 51.0 months, which was significantly greater than the 31.0 months observed in the high NLR (>1.77) group (P = 0.008). A significant difference in the median OS was also observed between the low (≤12.31) and high (>12.31) NMR groups (43.0 vs. 31.0 months, respectively; P = 0.042). Patients with low (≤40.0 g/L) albumin levels had a shorter survival time than patients with high (>40.0 g/L) albumin levels (26.0 vs. 42.0 months, respectively; P = 0.024; Fig 2). Other factors, including weight loss, tumor length, pathological T stage, pathological N stage, and pathological TNM stage, were also significantly correlated with OS (Table 2). In the multivariate analysis, which included factors identified as significant in the univariate analysis, the pathological TNM stage (hazard ratio (HR), 1.967; 95% CI, 1.392–2.779; P<0.001), weight loss (HR, 2.063; 95% CI, 1.200–3.545; P = 0.009), and the NLR (HR, 2.030; 95% CI, 1.262–3.264; P = 0.003) were all found to be independent prognostic factors for OS (Table 3).A further stratified analysis indicated that the NLR remained a strong prognostic indicator for OS in patients with stage I/II disease (Fig 3A). Our data suggest that the NLR is superior to the NMR and albumin levels in predicting BSCCE OS.

**Table 2:** Univariate analysis of prognostic factors for overall survival and recurrence-free survival in 121 patients with basaloid squamous cell carcinoma of the esophagus

		Overall Survival		Recurrence-free Survival	
		Median(mon)	P value	Median(mon)	P value
**Age(yr)**	≤60	38.0	0.463	26.0	0.750
	>60	32.0		23.0	
**Gender**	Male	38.0	0.195	23.0	0.224
	Female	26.0		18.0	
**Weight loss**	Yes	42.0	0.002	27.0	0.005
	No	24.0		7.0	
**Tumor location**	Upper	27.0	0.589	24.0	0.5
	Middle	35.0		18.0	
	Lower	38.0		26.0	
**Tumor length**	≤4.25	59.0	0.003	44.0	<0.001
**(cm)**	>4.25	26.0		10.0	
**Pathological T**	T1/2	51.0	0.008	39.0	0.006
**stage**	T3/4	28.0		15.0	
**Pathological N**	N-	43.0	0.001	27.0	0.008
**stage**	N+	24.0		14.0	
**Pathological**	I	NR	<0.001	NR	<0.001
**TNM stage**	II	38.0		22.0	
	III	23.0		9.0	
**Lymphocyte**	≤2.13	38.0	0.584	25.0	0.511
**count (×109/L)**	>2.13	36.0		21.0	
**Neutrophil**	≤3.74	42.0	0.181	27.0	0.097
**count (×109/L)**	>3.74	32.0		18.0	
**Monocyte**	≤0.27	32.0	0.387	21.0	0.870
**count (×109/L)**	>0.27	38.0		24.0	
**NMR**	≤12.31	43.0	0.042	27.0	0.149
	> 12.31	31.0		18.0	
**NLR**	≤1.77	51.0	0.008	44.0	0.002
	> 1.77	31.0		14.0	
**Albumin (g/L)**	≤40.0	26.0	0.024	12.0	0.115
	> 40.0	42.0		27.0	

NMR: neutrophil-to-monocyte ratio; NLR: neutrophil-to-lymphocyte ratio; NR: not reached.

**Table 3:** Cox proportional hazards model of prognostic factors for overall survival and recurrence-free survival in 121 patients with basaloid squamous cell carcinoma of the esophagus

	Multivariate analysis	
	HR(95%CI)	P value
**Overall Survival**		
**Pathological TNM stage, I vs II vs III**	1.967(1.392–2.779)	< 0.001
**Weight loss, Yes vs No**	2.063(1.200–3.545)	0.009
**NLR, ≤1.77 vs > 1.77**	2.030(1.262–3.264)	0.003
**Recurrence-free Survival**		
**Tumor length(cm), ≤4.25 vs >4.25**	1.868(1.138–3.064)	0.013
**Pathological TNM stage, I vs II vs III**	1.817(1.299–2.542)	< 0.001
**NLR, ≤1.77 vs > 1.77**	2.222(1.407–3.508)	0.001

NMR: neutrophil-to-monocyte ratio; NLR: neutrophil-to-lymphocyte ratio; HR: hazard ratio;TNM: tumor-node-metastasis.

### RFS analysis

The 1-, 3-, and 5-year RFS rates were 62.7%, 38.7%, and 28.1%, respectively. The median RFS was 23.0 months. The preoperative NLR was significantly associated with RFS; the median RFS was 44.0 months for the low (≤ 1.77) NLR group and 14.0 months for the high (> 1.77) NLR group (P = 0.002; Fig 2). However, other factors, including the NMR and albumin levels, were not significant predictors of RFS (Table 2). In the multivariate analysis, tumor length (HR, 1.868; 95% CI, 1.138–3.064; P = 0.013), TNM stage (HR, 1.817; 95% CI, 1.299–2.542; P<0.001), and the NLR (HR, 2.222; 95% CI, 1.407–3.508; P = 0.001) were all found to be independent prognostic factors for RFS (Table 3 and Fig 3). Furthermore, a preoperative NLR > 1.77 was an indicator of a relatively poor RFS for BSCCE stage I/II and stage III patients (Fig 3B and 3D).

## Discussion

The role of the preoperative NLR in prognosis prediction for BSCCE remains unclear. The present study has shown that a higher preoperative NLR is significantly associated with a poorer OS and RFS in BSCCE patients. We also separately validated the predictive value of the NLR in stage I/II and stage III patients. In this analysis, other immune and nutrition-related factors, including the preoperative lymphocyte count, neutrophil count, monocyte count, NMR, and albumin levels, were not prognostic factors for BSCCE. Our data suggest that, among these factors, the NLR is the best immune signature to predict survival. To the best of our knowledge, it is seldomly reported before that the NLR is an independent prognostic factor for patients with BSCCE.

The histopathological characteristics of BSCCE differ from typical SCC of the esophagus. In our pathological analysis of BSCCE samples, the typical microscopic features were a nesting, lobular, or trabecular arrangement of basaloid cells accompanied by comedo-type necrotic foci and small cystic spaces in the center of the tumor. It has been reported that BSCCE is accompanied by various histopathological components, including ductal differentiation, myoepithelial cells, and in some cases, adenoid cystic carcinoma-like features [4, 18, 19]. Immunohistochemical analyses have also shown immunoreactivity for a number of cytokeratin subtypes, bcl-2, and neuroendocrine markers, which could assist in the diagnosis and distinction of BSCCE. However, none of these markers are specific for BSCCE [2, 19–21]. The expression of Ki-67 is reported to be higher in BSCCE than in typical esophageal SCC, and telomerase activity has been detected in 95% BSCCE patients[22]. Several studies have found that BSCCE has enhanced proliferative activity and apoptotic indices, and is biologically more aggressive than typical SCC[23, 24]. Baba et al. reported that BSCCE and typical SCC show distinct genetic and epigenetic alterations, such as PIK3CA mutation and LINE-1 methylation[17]. Saito et al. [25] observed that the expression of sFRP-2 is reduced or absent in 90% of BSCCE cases, while the expression is observed in typical SCC and the normal esophageal membrane. Given all of these observations, we believe that BSCCE should be considered as a distinct entity, and the relationship between the preoperative NLR and BSCCE prognosis deserves further investigation.

The preoperative NLR has been reported to be a valuable prognostic factor in many carcinomas[12, 14, 15]. In esophageal carcinoma, Sharaiha et al. [16] demonstrated that a high preoperative NLR is predictive of a poor disease-free survival and OS. In another study [15], the pretreatment NLR was superior to the platelet-lymphocyte-ratio in predicting the long-term survival of locally advanced esophageal carcinoma patients treated with neoadjuvant chemotherapy and surgery. In two recent meta-analyses, which included 1633 and 1540 patients, the NLR was reported to be inversely correlated with the prognosis of esophageal carcinoma patients, and a high NLR was associated with an advanced tumor and node stage[26, 27]. Toyokawa et al. [28] have shown that esophageal cancer patients’ combined serum albumin concentration, peripheral lymphocyte count, and total cholesterol concentration can reflect their long-term survival. Our study exclusively evaluated the prognostic value of inflammatory markers in BSCCE. Using univariate analysis, we found that the preoperative NLR, NMR, and albumin level were significantly correlated with OS. However, in the subsequent multivariate analysis, only the NLR proved to be an independent prognostic indicator for BSCCE, with an AUC of 0.612. Similarly, the NLR was also an independent predictor for RFS, as were the TNM stage and tumor length.

Growing evidence has suggested a strong link between an individual’s inflammatory response and carcinoma. The association between the NLR and long-term cancer survival is complex and intriguing. Peripheral neutrophils and lymphocytes play a crucial role in the systemic inflammatory response, and so, they are considered a surrogate biomarker of an individual’s immune status. Neutrophils and lymphocytes can inhibit or promote cancer progression through microenvironment immune interactions. Tumor related chemokines, such as C-C motif chemokine ligand (CCL)4, CCL5, and CCL20, and C-X-C motif chemokine ligand 10 (CXCL10) can act as chemoattractants for immune cells, including CD8^+^ T cells, CD4^+^ T helper type 1 lymphocytes, CD4^+^ regulatory T cells, and natural killer cells [29, 30]. These tumor infiltrating lymphocytes are recruited into tumors, activated, and subsequently act to eliminate or inhibit cancer cells. High levels of CD8^+^ cytotoxic T cells and low levels of CD4^+^ regulatory T cells in the tumor microenvironment have been shown to positively correlate with a favorable clinical outcome in esophageal carcinoma[10, 29, 30]. Wang et al.[9] have demonstrated that the peritumoral neutrophil–to–CD8^+^ lymphocyte ratio is associated with an advanced T stage and lymph node metastasis in esophageal cancer. In the present study, although no significant correlation between the NLR and pathological N, pathological T, or TNM stage was identified, patients in the high NLR group had significantly more > 5.00 cm tumors. Furthermore, relative lymphocytopenia (lymphocyte count ≤2.13×10^9^/L) was observed in the high NLR group, which could reflect an immunosuppressive status that would favor tumor development and metastasis.

Many studies have tried to identify more valuable prognostic factors for esophageal cancer; it is extremely important to be able to predict prognosis at the commencement of therapy. We believe that cancer progression is affected by both the cancer cells and the patient’s immune characteristics. In the present study, the NLR was identified as a novel immune parameter for predicting the prognosis of BSCCE after surgery. Further analysis showed that an elevated preoperative NLR sensitively predicted a relatively poor OS and disease-free survival in different BSCCE TNM stages. This finding is in accordance with previous studies in other tumor types[13, 14, 16, 26], indicating that the NLR may serve as a marker of an individual’s immune status. However, the optimal cutoff value for patients who underwent a curable esophagectomy ranged from 3.0 to 5.0 in previous studies [16, 28, 31, 32]. This difference may stem from immunological heterogeneity in esophageal SCC and adenocarcinoma patients, or may be due to the preoperative chemoradiotherapy that some of the participants received, which could have significantly affect the patient’s immunological status. Study biases may also exist due to the limited sample sizes and retrospective designs of these studies.

There are several potential limitations to the present study. There is inevitable selection bias in the survival analysis because of the retrospective nature of the analysis. However, given that BSCCE is such a rare disease, only a small number of cases could be collected from a single institution. Biases are also inherent to multicenter analyses because the diagnostic and treatment procedures in different cancer centers vary. As such, further prospective studies with larger sample sizes are required to confirm the predictive value of the preoperative NLR in BSCCE.

In conclusion, our data show that the preoperative NLR could serve as a prognostic indicator for BSCCE patients following curative surgery. Considering that neutrophil count and lymphocyte counts are routinely determined prior to treatment, the NLR is a convenient and cost-effective biomarker for long-term survival analysis in BSCCE. The mechanisms underlying the relationship between the preoperative NLR and cancer progression require further investigation.

## Supporting Information

S1 File Patients' data are listed in detail in this file. (XLS)

## References

[1] Wain SL, Kier R, Vollmer RT, Bossen EH. Basaloid-squamous carcinoma of the tongue, hypopharynx, and larynx: report of 10 cases. Hum Pathol, 1986 17(11): 1158–1166. 3770734 10.1016/s0046-8177(86)80422-1

[2] Tsang WY, Chan JK, Lee KC, Leung AK, Fu YT. Basaloid-squamous carcinoma of the upper aerodigestive tract and so-called adenoid cystic carcinoma of the oesophagus: the same tumour type? Histopathology, 1991 19(1): 35–46. 1717358 10.1111/j.1365-2559.1991.tb00892.x

[3] Saito T, Mitomi H, Yao T. Molecular pathology and potential therapeutic targets in esophageal basaloid squamous cell carcinoma. Int J Clin Exp Pathol, 2015 8(3): 2267–73. 26045734 PMC4440043

[4] Li TJ, Zhang YX, Wen J, Cowan DF, Hart J, Xiao SY. Basaloid squamous cell carcinoma of the esophagus with or without adenoid cystic features. Arch Pathol Lab Med, 2004 128(10): 1124–30. 10.1043/1543-2165(2004)128<1124:BSCCOT>2.0.CO;2 15387711

[5] Sato-Kuwabara Y, Fregnani JH, Jampietro J, Carvalho KC, Franco CP, da Costa WL, et al Comparative analysis of basaloid and conventional squamous cell carcinomas of the esophagus: prognostic relevance of clinicopathological features and protein expression. Tumour Biol, 2016 37(5): 6691–6699. 10.1007/s13277-015-4551-3 26649862

[6] Zhang BH, Cheng GY, Xue Q, Gao SG, Sun KL, Wang YG, et al Clinical outcomes of basaloid squamous cell carcinoma of the esophagus: a retrospective analysis of 142 cases. Asian Pac J Cancer Prev, 2013 14(3): 1889–94. 23679289 10.7314/apjcp.2013.14.3.1889

[7] Fehrenbacher L, Spira A, Ballinger M, Kowanetz M, Vansteenkiste J, Mazieres J, et al Atezolizumab versus docetaxel for patients with previously treated non-small-cell lung cancer (POPLAR): a multicentre, open-label, phase 2 randomised controlled trial. Lancet, 2016 387(10030): 1837–1846. 10.1016/S0140-6736(16)00587-0 26970723

[8] Yang Y, Pang Z, Ding N, Dong W, Ma W, Li Y, et al The efficacy and potential predictive factors of PD-1/PD-L1 blockades in epithelial carcinoma patients: a systematic review and meta analysis. Oncotarget, 2016 8 15. [Epub ahead of print] 10.18632/oncotarget.11291 PMC5342058 27542277

[9] Wang J, Jia Y, Wang N, Zhang X, Tan B, Zhang G, et al The clinical significance of tumor-infiltrating neutrophils and neutrophil-to-CD8+ lymphocyte ratio in patients with resectable esophageal squamous cell carcinoma. J Transl Med, 2014 12: 7 10.1186/1479-5876-12-7 24397835 PMC3895663

[10] Lv L, Pan K, Li XD, She KL, Zhao JJ, Wang W, et al The accumulation and prognosis value of tumor infiltrating IL-17 producing cells in esophageal squamous cell carcinoma. PLoS One, 2011 6(3): e18219 10.1371/journal.pone.0018219 21483813 PMC3069054

[11] Salama P, Phillips M, Grieu F, Morris M, Zeps N, Joseph D, et al Tumor-infiltrating FOXP3+ T regulatory cells show strong prognostic significance in colorectal cancer. J Clin Oncol, 2009 27(2): 186–192. 10.1200/JCO.2008.18.7229 19064967

[12] Zhao W, Wu Z, Li Y, Jia H, Chen M, Gu X, et al Pretreatment neutrophil-to-lymphocyte ratio and its dynamic changes are associated with the overall survival in advanced cancer patients undergoing palliative care. Sci Rep, 2016 6: 31394 10.1038/srep31394 27510632 PMC4980771

[13] Wang SC, Chou JF, Strong VE, Brennan MF, Capanu M, Coit DG. Pretreatment Neutrophil to Lymphocyte Ratio Independently Predicts Disease-specific Survival in Resectable Gastroesophageal Junction and Gastric Adenocarcinoma. Ann Surg, 2016 263(2): 292–297. 10.1097/SLA.0000000000001189 25915915 PMC4905761

[14] Yuan D, Zhu K, Li K, Yan R, Jia Y, Dang C. The preoperative neutrophil-lymphocyte ratio predicts recurrence and survival among patients undergoing R0 resections of adenocarcinomas of the esophagogastric junction. J Surg Oncol, 2014 110(3): 333–340. 10.1002/jso.23651 24889121

[15] Ji WH, Jiang YH, Ji YL, Li B, Mao WM. Prechemotherapy neutrophil: lymphocyte ratio is superior to the platelet: lymphocyte ratio as a prognostic indicator for locally advanced esophageal squamous cell cancer treated with neoadjuvant chemotherapy. Dis Esophagus, 2016 29(5): 403–411. 10.1111/dote.12322 25625421

[16] Sharaiha RZ, Halazun KJ, Mirza F, Port JL, Lee PC, Neugut AI, et al Elevated preoperative neutrophil:lymphocyte ratio as a predictor of postoperative disease recurrence in esophageal cancer. Ann Surg Oncol, 2011 18(12): p. 3362–3369. 10.1245/s10434-011-1754-8 21547702 PMC3192937

[17] Baba Y, Ishimoto T, Harada K, Kosumi K, Murata A, Miyake K, et al Molecular Characteristics of Basaloid Squamous Cell Carcinoma of the Esophagus: Analysis of KRAS, BRAF, and PIK3CA Mutations and LINE-1 Methylation. Ann Surg Oncol, 2015 22(11): 3659–3665. 10.1245/s10434-015-4445-z 25691283

[18] Hamilton SR, Aaltonen LA. World Health Organization Classification of Tumours Pathology and Genetics of Tumours of the Digestive System. Lyon: IARC Press, 2000.

[19] Tsubochi H, Suzuki T, Suzuki S, Ohashi Y, Ishibashi S, Moriya T, et al Immunohistochemical study of basaloid squamous cell carcinoma, adenoid cystic and mucoepidermoid carcinoma in the upper aerodigestive tract. Anticancer Res, 2000 20(2B): 1205–1211. 10810423

[20] Cho KJ, Jang JJ, Lee SS, Zo JI. Basaloid squamous carcinoma of the oesophagus: a distinct neoplasm with multipotential differentiation. Histopathology, 2000 36(4): 331–340. 10759947 10.1046/j.1365-2559.2000.00851.x

[21] Huang Z, Shen Y, Liang Y, Wu X. Basaloid squamous cell carcinoma of the esophagus: an immunohistochemical study of 8 cases. Chin Med J (Engl), 2001 114(10): 1084–1088. 11677772

[22] Lam KY, Law S, Luk JM, Wong J. Oesophageal basaloid squamous cell carcinoma: a unique clinicopathological entity with telomerase activity as a prognostic indicator. J Pathol, 2001 195(4): 435–442. 10.1002/path.984 11745675

[23] Imamhasan A, Mitomi H, Saito T, Hayashi T, Takahashi M, Kajiyama Y, et al Immunohistochemical and oncogenetic analyses of the esophageal basaloid squamous cell carcinoma in comparison with conventional squamous cell carcinomas. Hum Pathol, 2012 43(11): 2012–2023. 10.1016/j.humpath.2012.02.010 22607702

[24] Kumagai Y, Nagata K, Ishiguro T, Haga N, Kuwabara K, Sobajima J, et al Clinicopathologic characteristics and clinical outcomes of esophageal basaloid squamous carcinoma: experience at a single institution. Int Surg, 2013 98(4): 450–454. 10.9738/CC195 24229040 PMC3829080

[25] Saito T, Mitomi H, Imamhasan A, Hayashi T, Mitani K, Takahashi M, et al Downregulation of sFRP-2 by epigenetic silencing activates the beta-catenin/Wnt signaling pathway in esophageal basaloid squamous cell carcinoma. Virchows Arch, 2014 464(2): 135–143. 10.1007/s00428-014-1538-1 24464051

[26] Yang X, Huang Y, Feng JF, Liu JS. Prognostic significance of neutrophil-to- lymphocyte ratio in esophageal cancer: a meta-analysis. Onco Targets Ther, 2015 8: 789–794. 10.2147/OTT.S77099 25914549 PMC4401207

[27] Yodying H, Matsuda A, Miyashita M, Matsumoto S, Sakurazawa N, Yamada M, et al Prognostic Significance of Neutrophil-to-Lymphocyte Ratio and Platelet-to-Lymphocyte Ratio in Oncologic Outcomes of Esophageal Cancer: A Systematic Review and Meta-analysis. Ann Surg Oncol, 2016 23(2): 646–654. 10.1245/s10434-015-4869-5 26416715

[28] Toyokawa T, Kubo N, Tamura T, Sakurai K, Amano R, Tanaka H, et al The pretreatment Controlling Nutritional Status (CONUT) score is an independent prognostic factor in patients with resectable thoracic esophageal squamous cell carcinoma: results from a retrospective study. BMC Cancer, 2016 16: 722 10.1186/s12885-016-2696-0 27599460 PMC5013653

[29] Liu J, Li F, Ping Y, Wang L, Chen X, Wang D, et al Local production of the chemokines CCL5 and CXCL10 attracts CD8+ T lymphocytes into esophageal squamous cell carcinoma. Oncotarget, 2015 6(28): 24978–2489. 10.18632/oncotarget.4617 26317795 PMC4694808

[30] Liu JY, Li F, Wang LP, Chen XF, Wang D, Cao L, et al CTL- vs Treg lymphocyte-attracting chemokines, CCL4 and CCL20, are strong reciprocal predictive markers for survival of patients with oesophageal squamous cell carcinoma. Br J Cancer, 2015 113(5): 747–755. 10.1038/bjc.2015.290 26284335 PMC4559838

[31] Feng JF, Huang Y, Chen QX. Preoperative platelet lymphocyte ratio (PLR) is superior to neutrophil lymphocyte ratio (NLR) as a predictive factor in patients with esophageal squamous cell carcinoma. World J Surg Oncol, 2014 12: 58 10.1186/1477-7819-12-58 24641770 PMC3973187

[32] Duan H, Zhang X, Wang FX, Cai MY, Ma GW, Yang H, et al Prognostic role of neutrophil-lymphocyte ratio in operable esophageal squamous cell carcinoma. World J Gastroenterol, 2015 21(18): 5591–5597. 10.3748/wjg.v21.i18.5591 25987784 PMC4427683

